# Exploring and expanding the phenotype and genotype diversity in seven Chinese families with spondylo-epi-metaphyseal dysplasia

**DOI:** 10.3389/fgene.2022.960504

**Published:** 2022-08-31

**Authors:** Shanshan Lv, Jiao Zhao, Li Liu, Chun Wang, Hua Yue, Hao Zhang, Shanshan Li, Zhenlin Zhang

**Affiliations:** ^1^ Shanghai Clinical Research Center of Bone Diseases, Department of Osteoporosis and Bone Diseases, Shanghai Jiao Tong University Affiliated Sixth People’s Hospital, Shanghai, China

**Keywords:** spondylo-epi-metaphyseal dysplasia, phenotype-genotype relation, TRPV4, COL2A1, CCN6, SBDS, ACAN

## Abstract

Spondylo-epi-metaphyseal dysplasia (SEMD) is a heterogeneous group of disorders with different modes of inheritance and is characterized by disproportionate or proportionate short stature. To date, more than 30 disease-causing genes have been identified, and different types of SEMD exhibit greatly overlapping clinical features, which usually complicate the diagnosis. This study was performed to expand the clinical and molecular spectrum of SEMD among Chinese subjects and to explore their potential phenotype–genotype relations. We enrolled seven families including 11 affected patients with SEMD, and their clinical, radiographic, and genetic data were carefully analyzed. All the seven probands showed different degrees of short stature, and each of them exhibited additional specific skeletal manifestations; four probands had extraosseous manifestations. X-rays of the seven probands showed common features of SEMD, including vertebral deformities, irregular shape of the epiphysis, and disorganization of the metaphysis. Seven variants were identified in *TRPV4* (c.694C> T, p.Arg232Cys), *COL2A1* (c.654 + 1G > C; c.3266_3268del, p.Gly1089del), *CCN6* (c.396 T> G, p.Cys132Trp; c.721 T>C, p.Cys241Arg), SBDS (c.258 + 2T> C), and *ACAN* (c.1508C> A, p.Thr503Lys) genes, and two of them were novel. Two families with *TRPV4* variants showed considerable intrafamily and interfamily heterogeneities. In addition, we reported one case of SEMD with a severe phenotype caused by *ACAN* gene mutation. Our study expands the phenotype and genetic spectrum of SEMD and provides evidence for the phenotype–genotype relations, aiding future molecular and clinical diagnosis as well as procreative management of SEMD.

## Introduction

Spondylo-epi-metaphyseal dysplasia (SEMD) is a heterogeneous genetic disorder, involving vertebral, epiphyseal, and metaphyseal dysplasia, and is diagnosed based on clinical phenotype, radiographic examination, and molecular sequencing. The primary clinical feature is a different degree of short stature (may present with short limbs or short trunk), combined with specific orthopedic symptoms (such as developmental coxa vara and scoliosis). Epiphyseal dysplasia usually leads to early-onset osteoarthritis, mostly in weight-bearing joints (Briggs et al., 1995). Odontoid hypoplasia causes atlantoaxial instability with severe spinal compression problems (Miyoshi et al., 2004). Radiological features included platyspondyly, vertebral body irregularity, destruction of articular cartilage, and dysplasia of epiphysis and metaphysis (Spranger, 1989).

**FIGURE 1:**
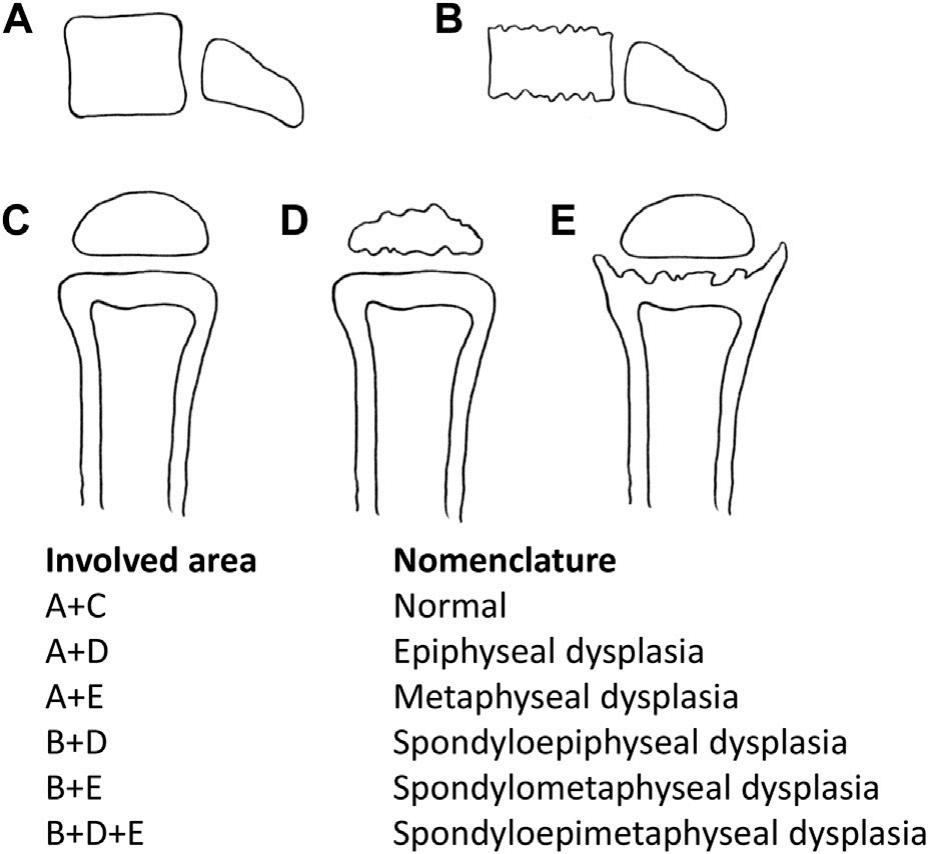
Nomenclature according to the involved area name of dysplasia. A + D: epiphyseal dysplasia, A + E: metaphyseal dysplasia, B + D: spondyloepiphyseal dysplasia, B + E: spondylometaphyseal dysplasia, and B + D + E: spondylo-epi-metaphyseal dysplasia.

This category of diseases is usually given the nomenclature according to the site name of manifest radiographic abnormalities (Alanay and Lachman, 2011; Mortier et al., 2019) (Figure 1), including spondyloepiphyseal dysplasia (SED), spondylometaphyseal dysplasia (SMD), spondylo-epi-metaphyseal dysplasia (SEMD), metaphyseal dysplasia (MD), and epiphyseal dysplasia (ED). With the in-depth study, SEMD is further classified according to specific clinical manifestations. For example, SED with different clinical manifestations can be divided into spondyloepiphyseal dysplasia congenita (SEDC, OMIM# 183900), spondyloepiphyseal dysplasia-Kimberley type (SED-KT, OMIM# 608361), and spondyloepiphyseal dysplasia-Maroteaux type (SED-MT, OMIM# 184095).

So far, more than 30 pathogenic genes have been identified to cause SEMD. These disease-causing genes are involved in encoding various types and functions of proteins (Cormier-Daire, 2008). It is difficult to define them as a common signal pathway among all the SEMD. In such clinical and genetic heterogeneous diseases, different gene–disease associations and overlapping clinical characteristics of different genes complicate the differential diagnosis. According to the nosology and classification of genetic skeletal disorders 2019 revision, groups 10–13 are classified as spondylo-epi-metaphyseal abnormalities, but these diseases still exist in other groups (Mortier et al., 2019). The more common SEMD were *COL2A1*-related dysplasia and pseudoachondroplasia (OMIM# 177170).

At present, most of the reports of SEMD are single case reports, and there are few studies focusing on exploring the phenotype of SEMD caused by different disease-causing genes. We reported seven families with SEMD caused by *TRPV4*, *COL2A1*, *CCN6*, *SBDS*, and *ACAN* genes in order to explore the relationship between phenotype and genotype of them. Several studies have reported the phenotype–genotype relations of skeletal disorders caused by *COL2A1*, *COMP*, and *CCN6* genes (Barat-Houari et al., 2016a; Madhuri et al., 2016; Liang et al., 2021), but we still need more families to prove it, especially Chinese families. Therefore, we summarized the clinical manifestations, radiological data, and molecular features of patients with SEMD, hoping to improve our understanding of Chinese families with SEMD.

## Methods

### Patients with clinical assessment

Seven non-consanguineous families (11 affected individuals) with features of spinal, epiphyseal, and metaphyseal dysplasia participated in this study from 2014–2020. Clinical data were collected, including basic information, family history, age of onset, physical examinations [included height, weight, arm, span, upper/lower segment (U/L) ratio, and standard deviation score (SDS) (Li, 2009)], and skeletal and other system manifestations. Radiographs of the spine, pelvises, and affected joints were taken. Laboratory examinations including liver and kidney functions, blood routine, and bone turnover markers were collected. The study was reviewed and approved by the Ethics Committee of the Shanghai Jiao Tong University Affiliated Sixth People’s Hospital and conducted in accordance with the Declaration of Helsinki. Informed written consents were obtained from all study participants or their legal guardians.

### Targeted exome sequencing

Peripheral blood samples were collected in EDTA tubes from seven probands and 20 available family members. Informed consent was obtained from all 27 participants before the genetic analysis. The QuickGene DNA whole blood kits (Kurabo Industries Ltd., Osaka, Japan) and the Nucleic Acid Isolation System (QuickGene-610L; Autogen, Inc., Holliston, MA, United States) were used to extract genomic DNA. We used the TES to detect candidate genes of seven probands’ DNA samples. TES was performed using the Agilent SureSelect (SureSelect Reagent Kit; Agilent Technologies, CA, United States) target enrichment kit, which consisted of 322 pathogenic genes known to cause skeletal disease (Lv et al., 2021). Sequencing was performed on the Illumina Hiseq platform using paired-end 150-bp reads.

### Evaluation of variants

We prioritized the variants that were known to cause disease in the Human Gene Mutation Database (HGMD) or other databases. The variants had lower allele frequency (< 0.001) in healthy control population databases (dbSNP, 1,000 Genomes, ESP6500, and gnomAD). The conservation and pathogenicity of variants were predicted by in silico tools (UniProt, Mutation Taster, Polyphen2, SIFT, and Human Splicing Finder). The pathogenicity of variants was also determined through appraisal of patients’ clinical phenotypes and scientific literature studies. The interpretation criteria of variants consisted of the American College of Medical Genetics and Genomics (ACMG) practice guidelines (Richards et al., 2015). Sanger sequencing was used to validate the candidate variants in all family members. Novel variants were recognized by using the Human Gene Mutation Database (HGMD) and ClinVar (Landrum et al., 2018; Stenson et al., 2020).

## Results

**FIGURE 2:**
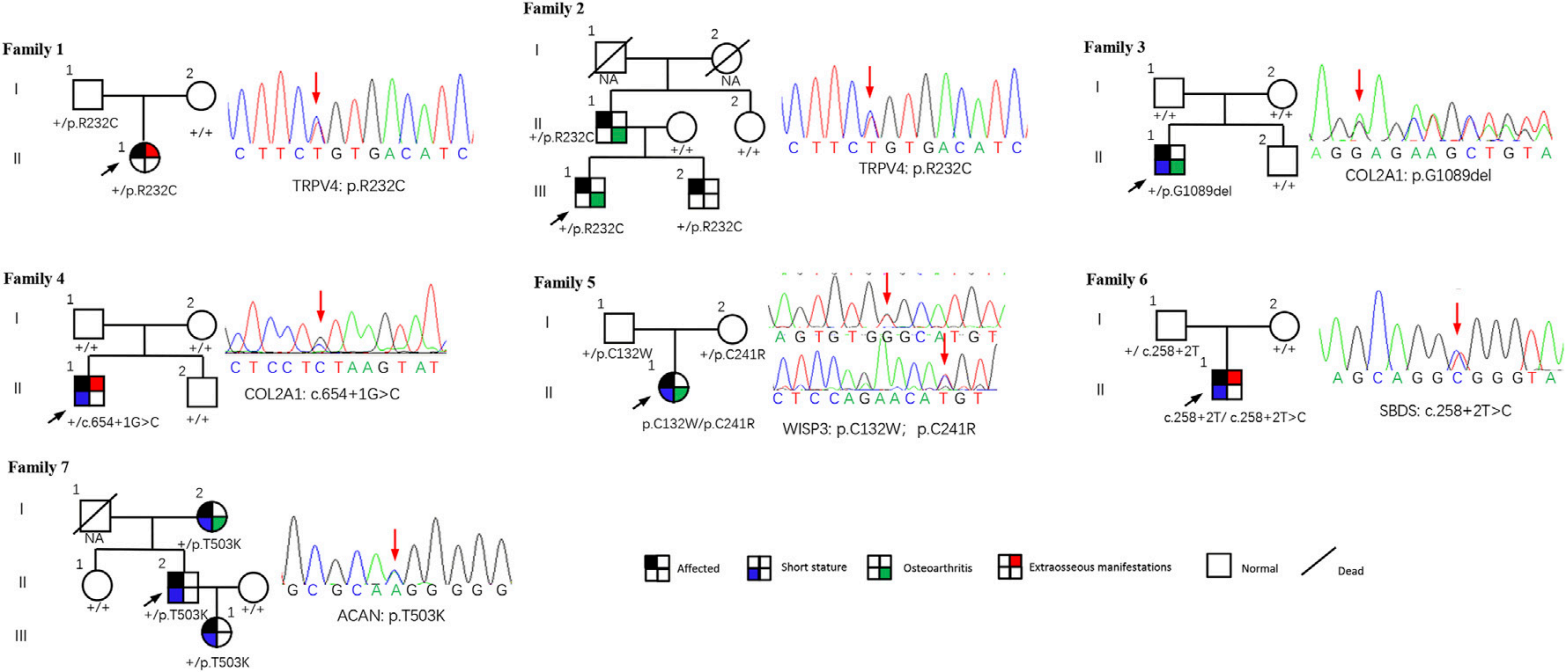
Pedigrees of seven families with SEMD. Almost all accessible family members were sequenced and examined. Mutations are shown below each subject, and the corresponding sequence diagrams are displayed next to the pedigrees. Circles and squares indicate females and males, respectively. Arrows identify the proband in the families. Slashes indicate deceased individuals.

This study included 11 patients derived from seven unrelated families who suffered from spinal, epiphyseal, and metaphyseal abnormalities. They are all of Han nationality from eastern Asia. Also, the ratio of females to males was 2/5, with 2 females and 5 males. The mean age of onset occurred at 4.9 ± 4.7 years. Detailed clinical characteristics of seven probands are shown in Table 1. Two families had an obvious positive family history, and the specific pedigrees of these seven families are shown in Figure 2.

**TABLE 1:** Clinical characteristics of seven probands with SEMD.

Patient	Gender	Age (y)	Age at onset	Weight (kg)	Height (cm)	SDS	Arm span	U/L	Muscle weakness	Osteoarthropathy	Joint contractures	Skeletal phenotype	Extraosseous phenotype	Diagnosis
P1	F	8.6	8	21	125	−1.1	118	63/62	No	No	No	Scoliosis	Myopia	Hereditary motor and sensory neuropathy
P2	M	13	10	41	155.4	−0.5	156	76/79.4	No	No	No	Lumbar lordosis, fourth metatarsal dysplasia (unusual feature)	No	Hereditary motor and sensory neuropathy
P3	M	8.7	0	37	101	−5.6	100	48/53	No	No	Yes	Severe short stature, pectus excavatum, scoliosis, and hip pain and stiffness	Tooth dysplasia	Spondyloepiphyseal dysplasia congenita
P4	M	29	0	40	146	−4.4	139	70/76	No	Yes	Yes	Severe short stature, enlargement extension of the fingers, elbows, knees, and ankles, and joint pain and stiffness	Retinal detachment and cataract	Kniest dysplasia
P5	F	14.6	10	44	154.9	−0.8	—	—	Yes	Yes	Yes	Enlargement extension of the fingers, elbows, knees, and ankles and joint pain and stiffness	No	Progressive pseudorheumatoid dysplasia
P6	M	11.8	6	39	116	−4.8	116	52/64	No	No	No	Severe short stature and joint pain	Mild intellectual disability and tooth dysplasia	Shwachman–Bodian–Diamond syndrome
P7	M	48	0	52	148	−4.0	135	—	No	Yes	No	Severe short stature, brachydactyly (unusual feature), clubfoot, and joint pain and stiffness	No	Spondyloepiphyseal dysplasia

P, probands; M, male; F, female; SDS, standard deviation score; U/L, upper /lower part of the body.

### *TRPV4*-related skeletal dysplasia (OMIM# 605427)

**FIGURE 3:**
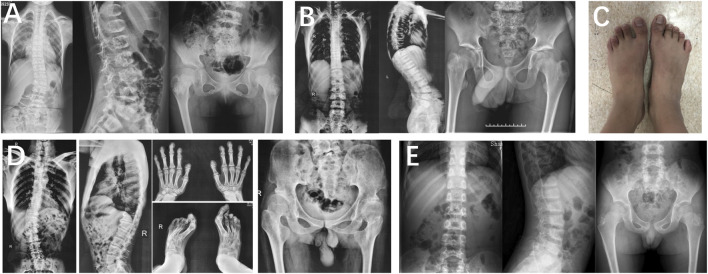
Photographs and radiographs of families 1 and 2 with TRPV4-related skeletal dysplasia. **(A)** Radiographs of the spine and pelvis of proband 1 (Ⅱ-1) in family 1. Mild scoliosis, ovoid vertebral bodies, and rough edges of the vertebral bodies were significant in the spine. Disorganization of metaphysis was in the pelvis. **(B,C)** Radiographs of the spine and pelvis **(B)**, and photographs of feet **(C)** of proband 2 (Ⅲ-1) in family 2. **(B)** Rough edges of the vertebral bodies and lumbar lordosis were visible in the spine. Disorganization of the metaphysis and shortening of the femoral neck were in the pelvis. **(C)** Fourth metatarsal dysplasia was in the left foot. **(D)** Radiographs of hands, spine, pelvis, and feet of proband 2’s father (Ⅱ-1). Mild contracture of the distal joints was shown in the hands. Scoliosis and depression of the anterior edge of vertebral bodies were displayed in the spine. Flattening of the acetabular roof, shortening of the femoral neck, and stenosis of joint spaces were in the pelvis. Hallux valgus deformity was seen in feet. **(E)** Radiographs of the spine and pelvis of proband 2’s brother (Ⅲ-2). Irregular shape of the vertebral bodies in the spine. Radiographs of the pelvis were normal.

In family 1, proband 1 (Ⅱ-1) was an 8.6-year-old girl with normal pregnancy, delivery, and family history. Her height was 125 cm (−1.1 SDS), and her arm span was 118 cm. She came to medical attention at the age of 8 when her mother noticed her scoliosis. Physical examinations revealed mild scoliosis, increased lumbar lordosis, and normal gait. She had myopia and normal hearing and intelligence. X-rays demonstrated mild scoliosis, ovoid vertebral bodies, rough edges of the vertebral bodies, and disorganization of the metaphysis (Figure 3).

Family 2 included three affected individuals, but their clinical phenotypes were highly variable. The 13-year-old boy proband 2 (Ⅲ-1) was 155.4 cm (−0.5 SDS) tall. Since the age of 10, there was a significant kyphosis in his lumbar without low back pain and joint movement limitation. Physical examinations revealed severe lumbar lordosis and fourth metatarsal dysplasia. His father (Ⅱ-1, 35 years old) had mild contracture of the distal joints of both hands and hallux valgus deformity of both feet since the age of 10 and difficulty in walking now. His brother (Ⅲ-1, 7 years old) did not show any abnormalities. Their heights were within the normal range; his father was 172 cm tall, and his brother was 123.5 cm tall. X-rays of the spine and pelvis in proband 2 showed rough edges of the vertebral bodies, lumbar lordosis, disorganization of the metaphysis, and shortening of the femoral neck. X-rays of the spine, pelvis, and feet of his father manifested scoliosis, depression of the anterior edge of vertebral bodies, flattening of the acetabular roof, shortening of the femoral neck, stenosis of joint spaces, and hallux valgus deformity (Figure 3). However, his brother’s X-ray showed a mild irregularity in the shape of the vertebral bodies.

### *COL2A1*-related skeletal dysplasia Spondyloepiphyseal dysplasia congenita (OMIM# 183900)

**FIGURE 4:**
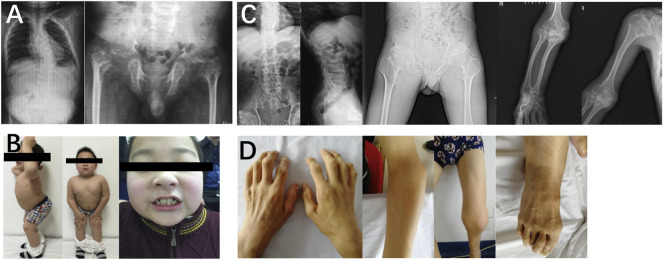
Photographs and radiographs of the families 3 and 4 with COL2A1-related skeletal dysplasia. **(A,B)** Radiographs of the spine and pelvis **(A)** and photographs of overall body and face **(B)** of proband 3 (Ⅱ-1) in family 3. **(A)** Scoliosis and platyspondyly were shown in the spine. Flattening and irregularity of the acetabular roof, femoral head necrosis, and disappearance of the femoral neck were visible in the pelvis. **(B)** Barrel chested and limited hip movement were performed in the overall body. No eruption of bilateral incisors was displayed in the face. **(C,D)** Radiographs of the spine, pelvis, and upper limb **(C)** and photographs of hands, elbows, knees, and feet **(D)** of proband 4 (Ⅱ-1) in family 4. **(C)** Severe multiple compressions of the vertebral bodies was revealed in the spine. Flattening of the acetabulum, shortening of the femoral neck, enlargement of the trochanter and femoral head, and narrow joint space of bilateral hips were shown in the pelvis. Widened epiphysis and metaphysis and joint cavity stenosis were visible in the elbows. **(D)** Bilateral elbow, knee, ankle, multiple metacarpophalangeal and interphalangeal joints were enlarged.

In family 3, proband 3 (Ⅱ-1) was a 9-year-old boy, with a height of 110 cm (−5.6 SDS). At 33 weeks of gestation, his mother’s color doppler ultrasound showed that the fetal femur was shortened by 1 cm. He had visible thoracic abnormalities (pectus excavatum) at birth, learned to walk at the age of 2, and underwent thoracic correction surgery at the age of 3. Since he was 6 years old, he had hip stiffness with pain and limited mobility. At present, he has been unable to straighten his waist. Physical examinations showed that both maxillary lateral incisors were absent. X-rays showed scoliosis, platyspondyly, flattening and irregularity of the acetabular roof, femoral head necrosis, and disappearance of the femoral neck (Figure 4).

### Kniest dysplasia (OMIM# 156550)

In family 4, the 29-year-old male proband 4 (Ⅱ-1) was 146 cm (−4.4 SDS) tall. He was born with enlarged knee joints, and thereafter, bilateral interphalangeal, elbow, knee, and ankle joints were enlarged gradually. At the age of 5, knee joint pain began and gradually aggravated, resulting in difficulty in walking. Proband 4 had poor visual acuity (retinal detachment in the left eye at the age of 24, cataract in the right eye at the age of 25, and both underwent surgery) and normal hearing. X-rays displayed severe multiple compressions of the vertebral bodies, flattening of the acetabulum, shortening of the femoral neck, narrow joint space of bilateral hips, and enlargement of the trochanter and femoral head (Figure 4).

### Progressive pseudorheumatoid dysplasia (OMIM# 208230)

**FIGURE 5:**
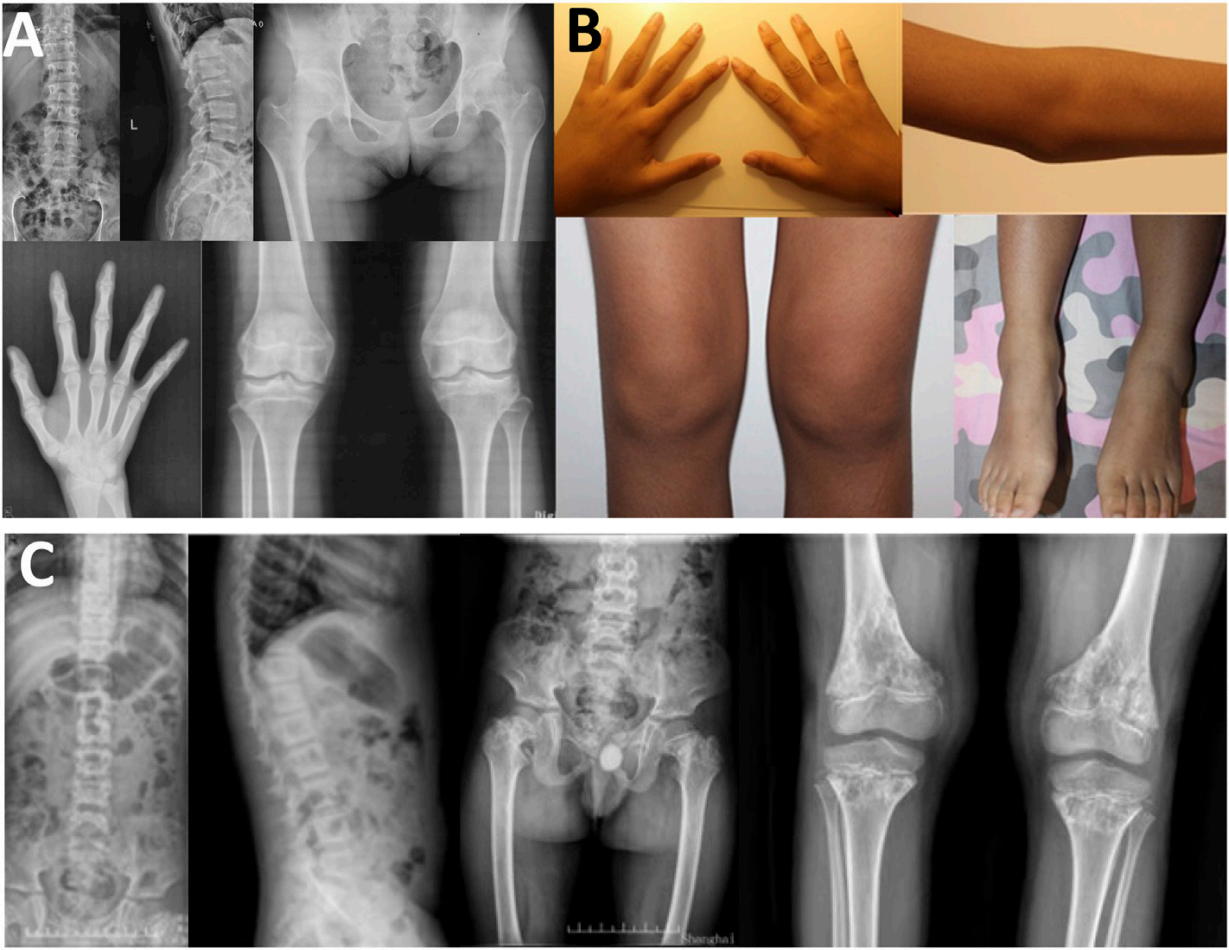
Photographs and radiographs of the families 5 and 6. **(A,B)** Radiographs of the spine, pelvis, hands, knees **(A)** and photographs **(B)** of proband 5 (Ⅱ-1) in family 5. **(A)** Mild platyspondyly was revealed in the spine. The pelvis was normal. Expansion of metaphysis and stenosis of joint spaces were visible in hands. Widened epiphysis and joint cavity stenosis were displayed in the knees. **(B)** Bilateral elbow, knee, ankle, and multiple metacarpophalangeal and interphalangeal joints were enlarged. **(C)** Radiographs of the spine, pelvis, and knees of proband (Ⅱ-1) in family 6. Irregular shape of the vertebral bodies was significant in the spine. Flattening of the acetabular roof, shortening of the femoral neck, mildly compressed femoral head, and thickening of metaphyseal texture were displayed in the pelvis. Widened epiphysis and metaphysis, irregular shape of the epiphysis, and disorganization of the metaphysis were obvious in the knees.

In family 5, the female proband 5 (Ⅱ-1) was 15 years old, with a height of 154.9 cm (−0.8 SDS). Intermittent large joint pain occurred at the age of 10, and no obvious abnormality was found in physical examinations. At the age of 13, the extension of both elbows was limited, and the elbow, knee, ankle, metacarpophalangeal, and interphalangeal joints gradually enlarged. Physical examinations showed that extension of multiple joints was limited, and no extraosseous manifestations were found. Symptoms aggravated with age. X-rays revealed mild platyspondyly, expansion of the epiphysis, and joint cavity stenosis. The pelvis was normal (Figure 5).

### Shwachman–Bodian–Diamond syndrome (OMIM# 260400)

In family 6, the male proband 6 (Ⅱ-1) was 12 years old and had a height of 116 cm (−4.8 SDS). He was born prematurely, and growth retardation occurred at birth. He developed knee pain at the age of 8, which was aggravated after exercise. Physical examinations revealed mild intellectual disability, tooth dysplasia, and normal gait. X-rays demonstrated irregular shape of the vertebral bodies and epiphysis, expansion of metaphysis, and disorganization of metaphysis (Figure 5).

### Aggrecan-related spondyloepiphyseal dysplasia (OMIM# 608361)

**FIGURE 6:**
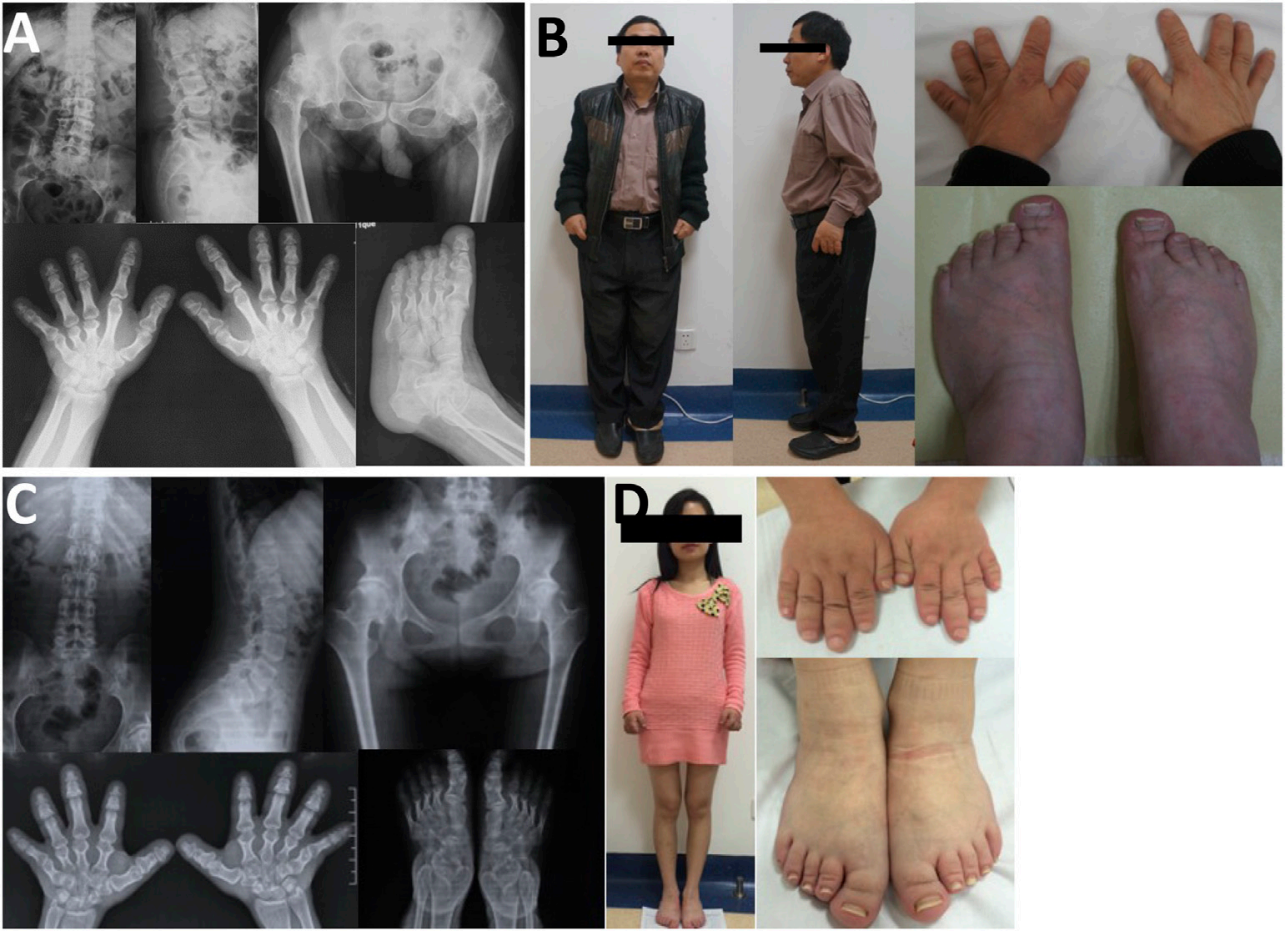
Photographs and radiographs of the family 7. **(A,B)** Radiographs of the spine, pelvis, hands, and feet **(A)** and photographs **(B)** of proband 7 (Ⅱ-2) in family 7. **(A)** Mild flattening and irregular shape of the vertebral bodies and 4–5 lumbar spondylolisthesis were shown in the spine. Flattening of the acetabulum, hip subluxation, femoral head necrosis, and shortening of the femoral neck were revealed in the pelvis. Widened epiphysis and joint cavity stenosis were displayed in the hands and feet. **(B)** Short stature with short limb was visible in the full body. Thick and short fingers and toes were shown in hands and feet. **(C,D)** Radiographs of the spine, pelvis, hands, and feet **(C)** and photographs **(D)** of patient (Ⅲ-1) in family 7. **(C)** Mild flattening and irregular shape of the vertebral bodies were displayed in the spine. The pelvis was normal. Widened epiphysis and joint cavity stenosis were displayed in the hands and feet. **(D)** Thick and short fingers and toes were shown in hands and feet.

In family 7, the 48-year-old male proband 7 (Ⅱ-2) had a height of 148 cm (−4.0SDS) and an arm span of 135 cm, with a positive family history. Brachydactyly, clubfoot, and growth retardation were found at birth. When learning to walk at the age of 1, he had mild knee valgus and a swinging gait. Pain in the spine, hip, and knee joints appeared at the age of 16, and symptoms worsened after 20 years. Physical examinations revealed stubby fingers and toes, mild knee valgus, and limited hip mobility. X-rays displayed mild flattening and irregular shape of the vertebral bodies, 4–5 lumbar spondylolisthesis, flattening of the acetabulum, hip subluxation, femoral head necrosis, shortening of the femoral neck, osteoarthritis, widened epiphysis, and joint cavity stenosis (Figure 6). Both his mother (Ⅰ-2, 73 years old, 150 cm, −2.0 SDS) and daughter (Ⅲ-1, 22 years old, 148 cm, −2.3 SDS) had similar symptoms: short stature with short limbs. Her daughter’s X-ray reports showed similar findings, but her pelvis was normal.

### Genetic analysis

We have identified *TRPV4, COL2A1, CCN6, SBDS*, and *ACAN* gene mutations in 8 p.R232C (c.694C> T) were detected in two probands and three family members. In family 3, a heterozygous deletion mutation in *COL2A1*, p.G1089del (c.3266_3268del), was detected in proband 3. In family 4, a heterozygous splicing mutation in *COL2A1*, c.654+1G > C, was detected in proband 4. In family 5, two missense mutations in *CCN6*, p.C132W (c.396 T>G) and p.C241R (c.721 T>C), were detected in proband 5. Furthermore, proband 5’s mother (c.721 T>C) and father (c.396 T>G) carried a mutation, respectively. In family 6, a homozygous splicing mutation in SBDS, c.258+2 T>C, was detected in proband 6. Also, we found a heterozygous splicing mutation (c.258+2 T>C) in proband 6’s mother. In family 7, a heterozygous missense mutation in *ACAN*, p.T503K (c.1508C> A), was detected in proband 7 and two affected family members. A total of seven variants were found, and the pathogenicity prediction and classification are shown in Table 2. Combined with literature studies, two variants (c.654 + 1G > C and p.T503K) were novel, and five variants (p.R232C, p.G1089del, p.C132W, p.C241R, and c.258+2 T>C) were reported previously.

**TABLE 2:** Description of the variants and predictions for pathogenicity.

Patient	Gene	RefSeq number	Mutation	Zygote	Segregation	Mutation Taster	Polyphen2	SIFT	CADD Phred	Evidence of classification	Classification	Reported
P1	*TRPV4*	NM_021625	c.694C>T and p.R232C	Heterozygous	Paternal	Disease causing	Probably damaging	Deleterious	31	PS1, PS3, PS4, PM2, PP2, and PP3	P	Yes
P2	*TRPV4*	NM_021625	c.694C>T and p.R232C	Heterozygous	Paternal	Disease causing	Probably damaging	Deleterious	31	PS1, PS3, PS4, PM2, PP2, and PP4	P	Yes
P3	*COL2A1*	NM_001844	c.3266_3268del and p.G1089del	Heterozygous	De novo	—	—	—	—	PS1, PM2, PM4, and PM6	P	Yes
P4	*COL2A1*	NM_001844	c.654+1G > C	Heterozygous	De novo	—	—	—	—	PVS1 and PM2	P	—
P5	*CCN6*	NM_198239	c.396 T>G and p.C132W	Compound heterozygous	Paternal and maternal	Disease causing	Probably damaging	Deleterious	24/28.4	PS1, PM2, PM3, PP4, and PP2; PS1, PS4, PM2, PM3, PP2, and PP4	P/P	Yes
			c.721 T>C and p.C241R		Paternal and maternal	Disease causing	Probably damaging	Deleterious				
P6	*SBDS*	NM_016038	c.258+2 T>C	Homozygous	Maternal and de novo	-	-	-	25.6	PVS1, PS1, PP1, and PP4	P	Yes
P7	*ACAN*	NM_001135	c.1508C>A and p.T503K	Heterozygous	Maternal	Disease causing	Probably damaging	Deleterious	24.5	PM2, PP1, PP3, PP2, and PP4	LP	-

P, pathogenic; LP, likely pathogenic.

## Discussion

This study focused on exploring phenotype and genotype in seven families with SEMD caused by *TRPV4*, *SBDS*, *COL2A1*, *CCN6*, and *ACAN* gene mutations, including two *TRPV4*-related skeletal dysplasia, one spondyloepiphyseal dysplasia, one Kniest dysplasia, one progressive pseudorheumatoid dysplasia, one Shwachman–Bodian–Diamond syndrome, and one aggrecan-related SED. The main clinical feature was short stature, and four probands had severe short stature (<-3 SDS). In addition, four probands had extraosseous manifestations. We have reported seven different mutation sites, including two novel sites and five reported sites. These findings expanded the phenotypic and genetic spectrum of SEMD in the Chinese population.

*TRPV4* gene encodes a nonselective calcium-permeable ion channel, widely distributed in bone, nerve, lung, heart, and other tissues (Everaerts et al., 2010). So far, more than 70 variants in the *TRPV4* gene had been reported to be related to autosomal-dominant skeletal dysplasia and motor and sensory neuropathies, such as Charcot-Marie-Tooth type 2C (CMT2C, OMIM#606482), scapuloperoneal spinal muscular atrophy (SPSMA, OMIM# 181405), spondylometaphyseal dysplasia-Kozlowski type (SMDK, OMIM#184252), and spondylo-epi-metaphyseal dysplasia Maroteaux pseudo-Morquio type 2 (SEDM-PM2, OMIM# 184095). The R232C variant had been reported repeatedly. Two studies reported that patients with R232C had a CMT2C-SPSMA overlap syndrome (Klein et al., 2011; Koutsis et al., 2015). They all had vocal cord paralysis, which was not found in this study. The probands in families 1 and 2 with R232C manifested slight short stature and scoliosis. Moreover, X-rays showed dysplasia of the vertebral bodies. No evidence of distal purely motor neuropathy was found in two probands, but these symptoms progressed very slowly. Therefore, we should follow up closely. Although only a small number of patients have been identified, they had comorbidity of neuromuscular and skeletal dysplasia (Cho et al., 2012; Faye et al., 2019). Interestingly, the clinical manifestations of family members who found the same variants showed intrafamily and interfamily variabilities. We detected that proband 1′s father harbored the R232C variant unexpectedly. He does not seem to be affected. Proband 2′s father and brother harbored the same variant. The phenotype of his father was the most serious, while his brother had no obvious abnormality, and only a mild irregularity in the shape of the vertebral bodies was found on the X-ray. These performances reinforced the notion that clinical features are heterogeneous in *TRPV4*-related neuropathies and skeletal dysplasia (Echaniz-Laguna et al., 2014). Meanwhile, the R232C variant exhibited reduced penetrance, which is the same as other reports (Chen et al., 2010; Koutsis et al., 2015). The R236C and R315W variants had also been reported to have reduced penetrance (Auer-Grumbach et al., 2010; Jędrzejowska et al., 2019). However, this phenomenon has not been reported in families with *TRPV4*-associated skeletal dysplasia (Nishimura et al., 2012).

The *COL2A1* gene encodes the alpha-1 chain of type II procollagen, which is the main component of the nucleus pulposus of intervertebral discs, the vitreous humor of the eyes, and hyaline cartilage extracellular matrix (Li et al., 1995). The phenotypic spectrum of *COL2A1*-related diseases is very broad and includes mainly spondyloepiphyseal dysplasia congenita (SEDC, OMIM#183900), Kniest dysplasia, spondyloperipheral dysplasia (SPPD, OMIM#271700), osteoarthritis with mild chondrodysplasia (OSCDP, OMIM# 604864), and spondyloepiphyseal dysplasia-Strudwick type (SED-ST, OMIM#184250). It encompasses a diverse group of clinical phenotypes characterized by short stature and ocular manifestations, whereas later it is manifested as isolated arthritis. SEDC is characterized by a short trunk, flattened vertebral bodies, and abnormal epiphyses. In our study, proband 3 had a short trunk (U/P = 0.91), severe hip joint mobility limitation, and femoral head necrosis. He had a deletion of glycine in Gly-X-Y repeats (p.Gly1089del), which led to severe impairment of protein assembly and stability (Barat-Houari et al., 2016b). Patients with C-propeptide glycine substitutions have been reported to be shorter than those with N-propeptide substitutions (Terhal et al., 2012). Our center has reported that glycine to serine substitution caused milder phenotypes than glycine to nonserine substitutions (Xu et al., 2020). In Kniest dysplasia, the classical phenotypes are short-trunk dwarfism due to severely affected skeletal growth, scoliosis, platyspondyly, and joint enlargement, while extraskeletal features mainly include myopia, prominent eyes, conductive hearing loss, and mid-face hypoplasia (Barat-Houari et al., 2016a). Proband 4 with Kniest dysplasia had severe short stature (−4.4 SDS, U/L = 0.92), severe skeletal deformity, retinal detachment and cataract, and normal face and hearing. It is worth mentioning that proband 4 was misdiagnosed as PPD before, but he was detected with a *COL2A1* mutation. This reminds us that we should pay attention to the differential diagnosis of PPD and *COL2A1*-related SEDC. The onset age of SEDC is usually at birth, while PPD mostly occurs at the age of 3–8 years (Garcia Segarra et al., 2012). At least 33 mutations were reported to cause Kniest dysplasia, and we reported a novel splice-site mutation (c.654 + 1G > C) which led to exon skipping.

Progressive pseudorheumatoid dysplasia (PPD) is a rare autosomal recessive disease caused by the functional loss or abnormality of cellular communication network factor 6 (*CCN6*). The clinical features are progressive joint stiffness and enlargement without inflammation (Dalal et al., 2012). Patients with PPD are usually normal at birth, with an onset ranging from 1 to 16 years of age. (Delague et al., 2005; Dalal et al., 2012). In our study, proband 5 developed symptoms at an average age of 10 years and were misdiagnosed as rheumatoid arthritis, but there was no inflammation. PPD initially presented with swelling and stiffness of the interphalangeal joint, gradually involving all large joints. According to literature statistics, the height of adults is usually less than the 3rd percentile (Garcia Segarra et al., 2012). In the early stages of the disease, the stature and proportion of patients may still be normal. Although patients had enlarged joints and limited mobility, X-rays showed no evidence of osteoarthritis. As cartilage destruction increases, secondary osteoarthritis may develop in adulthood. In our study, we found two reportedly missense mutations (C132W and C241R). The C241R variant has been reported many times in Chinese families and seems to be a hot site in Chinese (Ye et al., 2012; Yu et al., 2015; Hu et al., 2017). One research has reported that most PPD patients carry nonsense mutations in both *WISP3* alleles. They observed that the phenotype of patients homozygous for a missense mutation was not milder than patients with one or both alleles carrying a nonsense mutation (Garcia Segarra et al., 2012).

Shwachman–Bodian–Diamond syndrome (SBDS, OMIM#260400) is an autosomal recessive disease characterized by hematologic abnormalities, exocrine pancreatic dysfunction, immune deficiency, and skeletal abnormalities (Ginzberg et al., 1999). More than 90% of SBDS were caused by *SBDS* gene mutations (Boocock et al., 2003), and the most common variants were c.184A > T and c.258 + 2 T > C (Kuijpers et al., 2005; Nelson and Myers, 2018). The diagnosis of SBDS relied on evidence of exocrine pancreatic dysfunction, hematologic abnormalities, and recurrent infections (Huang and Shimamura, 2011; Nelson and Myers, 2018). However, some patients did not have this classic combination of manifestations (Myers et al., 2014). One study evaluated 102 genetically diagnosed patients with SBDS and found 40% had hematologic complications (Donadieu et al., 2012). According to research in China, the onset age of hemocytopenia ranges from 0 to 12 years old (An et al., 2020). In our study, proband 6 had normal hematological function. Therefore, it is necessary to conduct long-term follow-up on the hematologic complications of proband 6. Skeletal dysplasia is also a common manifestation, Mäkitie reported that the skeletal abnormalities in patients with SBDS varied not only with age but also with individuals (Mäkitie et al., 2004). Proband 6 had obvious skeletal manifestations, severe short stature (< −3.0 SDS), and epiphyseal and metaphyseal dysplasia. One study found that patients with SBDS ranged widely in their neurocognitive impairment compared with controls (Kerr et al., 2010) and had been shown to be related to structural brain alterations (Toiviainen-Salo et al., 2008). It shows that the phenotypic spectrum of SBDS is wider than previously thought. One study summarized the literature for large SBDS cohorts and found that no obvious genotype–phenotype correlation was observed (Mäkitie et al., 2004; Donadieu et al., 2012; Myers et al., 2020).

The cartilage aggrecan proteoglycan is encoded by the *ACAN* gene, the main structural component of the cartilage growth plate, which is essential for skeletal growth and articular cartilage function (Roughley and Mort, 2014). The mutations in the *ACAN* gene can be classified into four categories: spondyloepiphyseal dysplasia, Kimberley type (SEDK, OMIM#608361); spondylo-epi-metaphyseal dysplasia, aggrecan type (SEMD, OMIM#612813); osteochondritis dissecans (OCD, OMIM#165800); and short stature and advanced bone age. Previously, *ACAN* mutations had been reported as a cause of short stature with a frequency of 1.4–37.5% in the short stature population (Hauer et al., 2017; Stavber et al., 2020). In 2021, Li reported a large cohort of Chinese short-stature children caused by *ACAN* mutations (Lin et al., 2021). Patients with SEDK mostly have brachydactyly, platyspondyly, irregular femoral epiphyses, and precocious arthropathy, similar to the patients in this study. However, other features such as facial dysmorphisms and discopathy were not found. In contrast to previous data, brachydactyly was observed in all probands (Sentchordi-Montané et al., 2018). Nevertheless, there are few reports of SEDK caused by *ACAN* mutations (Gleghorn et al., 2005; Sentchordi-Montané et al., 2018). The new variant we reported expanded the clinical phenotype and genotype of this disease. At present, many clinical trials have proved that combined treatment with growth hormone (GH) or gonadotropin-releasing hormone (GnRH) is used to improve the final height of patients (Lin et al., 2021; Wei et al., 2021). Gkourogianni reported that the average height of patients treated with GH was lower than normal at 2.5 SD, while that of untreated patients was lower than 3 SD (Gkourogianni et al., 2017). The efficacy of GH and GnRH analogs in the treatment of patients with *ACAN* mutations needs more studies to be proved.

This study had some limitations. First, we were unable to perform electromyogram (EMG) testing in patients from family 1 and family 2 because the patients refused. We failed to determine whether the patients had manifestations of distal purely motor neuropathy. Second, as this was a single-center study, the number of probands with SEMD was small. Therefore, we did not have sufficient evidence to elucidate the phenotype–genotype relationship in SEMD. In the future, we hope to accumulate more patient data to illustrate the correlation between the two.

In the present study, we summarized seven families with SEMD caused by *TRPV4, COL2A1, CCN6, SBDS*, and *ACAN* gene mutations. Our results further demonstrated that phenotypic and genotypic spectra of SEMD are very wide. We should pay attention to the complications of SEMD and reduce or prevent their effects. Genetic testing is important for diagnosis, genetic counseling, and prenatal diagnosis. The new findings reported herein would contribute to the investigation of the phenotype–genotype relations among patients with SEMD.

## Data Availability

The datasets for this article are not publicly available due to concerns regarding participant/patient anonymity. Requests to access the datasets should be directed to the corresponding author.
